# Availability of age-stratified evidence for GLP1 receptor agonists and SGLT2 inhibitors: an analysis of European public assessment reports with a focus on older patients

**DOI:** 10.3389/fphar.2026.1786211

**Published:** 2026-10-20

**Authors:** Svea Stüdemann, Maik Gollasch, Stefan Engeli

**Affiliations:** 1 Department of Clinical Pharmacology, Institute of Pharmacology at the Center of Drug Absorption and Transport (C_DAT), University Medicine Greifswald, Greifswald, Germany; 2 Department of Internal Medicine and Geriatrics, University Medicine Greifswald, Greifswald, Germany

**Keywords:** chronic kidney disease, diabetes mellitus type 2, frailty, geriatrics, GLP1 receptor agonists, heart failure, obesity, old age

## Abstract

**Background:**

GLP1 receptor agonists and SGLT2 inhibitors are widely prescribed and their use is increasingly frequent in older patient populations. We evaluated the extent to which older patients were represented in clinical trials of liraglutide, dulaglutide, semaglutide, tirzepatide, dapagliflozin and empagliflozin.

**Methods:**

To identify relevant trials, we screened the respective European Public Assessment Reports (n = 28) and the European Summaries of Product Characteristics (n = 9) for each of the six approved medicines. Further information was retrieved from the original publications of the identified trials and from the ClinicalTrials.gov database (n = 40). Overall, we analyzed n = 185 sources for 106 trial populations.

**Results:**

For 46% of the 106 analyzed trial populations, data for patients ≥65 years were identified. Where age distributions were reported, patients aged 75 years or older were markedly underrepresented in nearly all trials, and subgroup analyses of efficacy and safety by age were rarely published. Especially in cardiovascular outcome trials and trials including patients with chronic kidney disease or chronic heart failure, older patients were better represented and analyses of primary outcomes in different age groups were published more frequently. Safety data on different age groups were mainly identified in the European Public Assessment Reports. Whereas increasing numbers of adverse events with older age were observed in several trials, this increase typically affected all trial arms, independent of the drug treatment.

**Conclusion:**

Data availability for patients with older age is insufficient from pivotal trials to properly describe efficacy or safety of liraglutide, dulaglutide, semaglutide, tirzepatide, dapagliflozin, or empagliflozin.

## 1 Introduction

Based on an impressive number of successful phase 3 trials, GLP1 (glucagon like peptide 1) receptor agonists (GLP1-RA) (Cooper and Van Raalte, 2025) and sodium-glucose cotransporter 2 inhibitors (SGLT2-I) (Hanke et al., 2025) are widely used drugs nowadays. Approved indications have constantly increased, and in the meantime comprise type 2 diabetes mellitus, obesity, heart failure, and chronic kidney disease. Several other experimental indications are currently under investigation.

The action of liraglutide, dulaglutide, and semaglutide is based on activating the GLP1 receptor exclusively, whereas tirzepatide is a dual GLP1 and GIP (glucose-dependent insulinotropic peptide) receptor agonist with a stronger weight reducing activity compared to pure GLP1-RA (Park et al., 2024). GLP1 receptors are expressed, for example, in pancreas, brain, heart, vasculature, kidney and immune system. GLP1-RA reduce blood glucose concentrations in a glucose-dependent manner. Also, gastric emptying is delayed, the feelings of satiety and fullness are sensed earlier and are more pronounced, craving for fatty food is decreased and enhanced control of eating behaviors occur. Besides glycemic control and weight reduction, clinical trials demonstrated a reduction of major adverse cardiovascular events (MACE), as well as improvements of systolic blood pressure and plasma lipid profile, and also anti-inflammatory effects, not the least on the level of the endothelium.

The inhibition of renal sodium-glucose cotransporter 2 (SGLT2) leads to a reduction of tubular glucose and sodium reabsorption with increased urinary excretion of glucose and osmotic diuresis resulting in increased tubuloglomerular feedback and diminished intraglomerular pressure (Parveen et al., 2025). Besides glycemic control, reductions of blood pressure, of cardiac pre- and afterload, and of volume overload, clinical trials demonstrated a reduction of major adverse cardiovascular events (MACE) including a reduced number of hospitalizations due to heart failure, as well as slowed decline in glomerular filtration rate (GFR), and reduced cardiovascular or renal death (Karagiannis et al., 2021).

In light of the broad indications and extensive prescribing of both drug classes, our objective was to assess the evidence supporting their use in older and very old patients, because they constitute a key population for GLP1-RA and SGLT2-I therapy. Whereas some evidence concerning efficacy has been reported previously (Karagiannis et al., 2021), adverse drug reactions (ADR) associated with the use of both drug classes in older patients are clearly underreported in the literature, although they are at particular risk to experience typical ADR associated with both drug classes. In Figure 1, we summarize the best described modes of action and clinical effects of GLP1-RA and SGLT2-I, and also well-known ADRs associated with their use which are mostly easily to explain by their modes of action. Whereas the significance of ADR such as volume depletion, hypotension, urinary tract infections, vulvovaginitis, and ketoacidosis in older patients are readily understood, weight loss - often a therapeutic goal with GLP1-RAs - may be problematic in older and very old patients.

**FIGURE 1:**
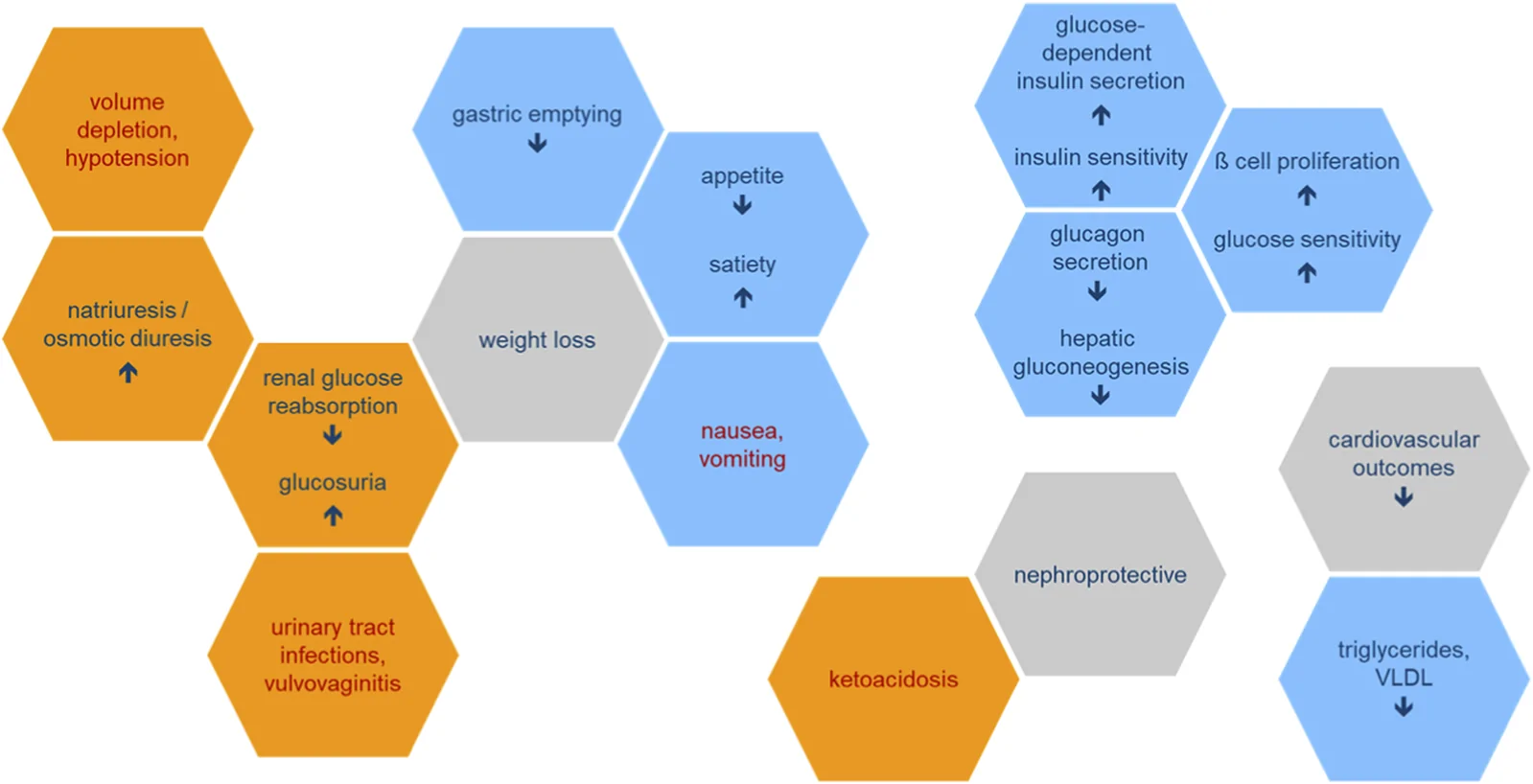
Positive effects of GLP1 receptor agonists (GLP1-RA, blue hexagons) and SGLT2 inhibitors (SGLT2-I, orange hexagons) are depicted in blue font, and known adverse drug reactions are depicted in red font.

First, unintentional weight loss in chronically ill patients, many of whom are old, is typically associated with a worse prognosis (Perera et al., 2021). Delaying gastric emptying in old patients might interact with the adherence to oral drug prescriptions (which are typically many in old age) but also might influence eating habits in a manner leading to malnutrition (Tzakri et al., 2024). Second, weight loss not only reduces fat mass but also skeletal muscle mass (Linge et al., 2024). The loss of both is highly variable, and depends on the mechanism of weight reduction and the concomitant approaches to body weight changes and other pharmacotherapies. While increasing physical activity can help to counteract muscle loss, this strategy becomes less effective as individuals grow older. Declining muscle mass is a characteristic aspect of aging, and frailty, a core geriatric syndrome, is particularly characterized by malnutrition and muscle wasting (Tsvetkov et al., 2025). These issues have been addressed in part in the literature, but mainly through discussions of the pathophysiology of muscle wasting, and by analyzing animal models and small experimental studies in humans (Chen et al., 2025; Ceasovschih et al., 2025; Bujdei-Tebeică et al., 2025).

A dedicated analysis of clinical outcomes from large phase 3 trials focusing on older and very old patients is lacking. Recent reviews discuss some aspects related to older patients, often with focus on specific indications like weight loss (Moscucci et al., 2026), heart failure (Khalid et al., 2026) as well as outcomes like cardiovascular or renal events (Waqas et al., 2025; Parrini et al., 2026; Gao et al., 2026), or effects on body composition (Simsek and Ucar, 2026; Joongpan et al., 2026; Scheen, 2026a; Scheen, 2026b). These reviews did not specifically address pivotal trials, or included uncontrolled observational studies, phase 4 trials or trials including substances which are not approved by EMA (Marassi and Fadini, 2026; Rego De Figueiredo et al., 2026; Kochanowska et al., 2026; Sridharan and Sivaramakrishnan, 2026; Omrani et al., 2026). To the best of our knowledge, no available review oversee a comparable large number of trials and patient data and/or focused on pivotal trials.

Here, we provide an analysis to address this gap, with particular emphasis on the representation of older patients in phase 3 pivotal trials of liraglutide, dulaglutide, semaglutide, tirzepatide, dapagliflozin and empagliflozin. The central objective of this review is to assess the extent to which older patients were included in the pivotal trials of GLP1-based therapies and SGLT2-I. The inclusion criteria of this review comprise the pivotal trials regardless of the included age groups to make conclusions about the availability or lack of age-stratified evidence. It is important to note that this review does not focus on randomized controlled trials in general, but specifically on pivotal trials. Pivotal trials are important and one of the primary sources that, in addition to the requirements for marketing authorization, for example, also have an impact on the content of summary of product characteristics (SmPC), are relevant to benefit assessments by statutory health insurers and influence physicians’ treatment decisions. Accordingly, especially for age-related diseases like heart failure or diabetes mellitus type 2, the evidence gathered in pivotal trials regarding the use of drugs in older adults is of particular interest.

## 2 Methods

Our analysis encompasses all indications that had received regulatory approval in Europe by July 2025 for the active compounds liraglutide, dulaglutide, semaglutide, tirzepatide, dapagliflozin and empagliflozin. For this purpose, the European Public Assessment Reports (EPAR) of the European Medicines Agency (EMA) as well as the Summary of Product Characteristics (SmPC) of these six drugs were analyzed as a starting point to identify phase 3 trials and their respective results relevant for the approval process. As “trial” we defined a clinical study with a specific study adult population characterized by inclusion and exclusion criteria and an entry into a publicly accessible database. We focused on phase 3 trials because they deliver the most relevant information to answer our research questions. Publications of these trials were accessed via PubMed to complement the EPAR data. We did not perform any additional PubMed research on any other search string than the individual trial name or number. Additionally, the entries of these trials in ClinicalTrials.gov were screened for information regarding the age of the trial population and age-related outcomes when EPAR, SmPC, and trial publications were not sufficient to answer our research questions. For some trials, more than one source was identified to include information regarding that trial. In this case, we analyzed all of these sources, because sometimes, the content regarding one trial varied considerably between the above-mentioned sources.

The sources for age-related information on trial populations and the full-text analyses of EPAR, SmPC, trial publications, and ClinicalTrials.gov entries (as applicable) were retrieved by one and controlled by a second author. No data extraction mistakes were discovered by the controlling author, and no disagreements occurred during data analyses and interpretation.

The included trials were screened for information about age composition of the trial populations like age as stated in the inclusion criteria, the mean age or proportion of different age groups (i.e., ≥ 65 years). Each trial was analyzed for age-specific reports on efficacy and safety. The analyzed sources mainly used the following age categorization: ≥65; ≥75 or ≥85 years. Sometimes however, a different categorization was used which we also reported.

## 3 Results

### 3.1 Selection process

This review is based on the analysis of n = 28 EPAR and n = 9 SmPC of the active compounds liraglutide, dulaglutide, semaglutide, tirzepatide, dapagliflozin and empagliflozin (Figure 2; Supplementary Tables S1, S2). By screening these primary sources, we identified n = 106 trials in the EPAR and n = 57 trials in the SmPC. Comparing both results, we excluded n = 57 duplicates. N = 106 trials were included for the analysis of the trial populations age composition, and age-related outcome reports. Information on these items were found in n = 145 sources, including the EPAR, SmPC and trial publications, because some trials were reported in more than one source. If there was a lack of information about the proportion of different age groups, we complemented our search with study-related data from ClinicalTrials.gov entries, as far as available. Overall, we included n = 185 sources related to the 106 trials. Whereas not all sources related to one specific trial included the identical set of data, numbers given for age proportions and age-related outcomes were identical if reported more than once.

**FIGURE 2:**
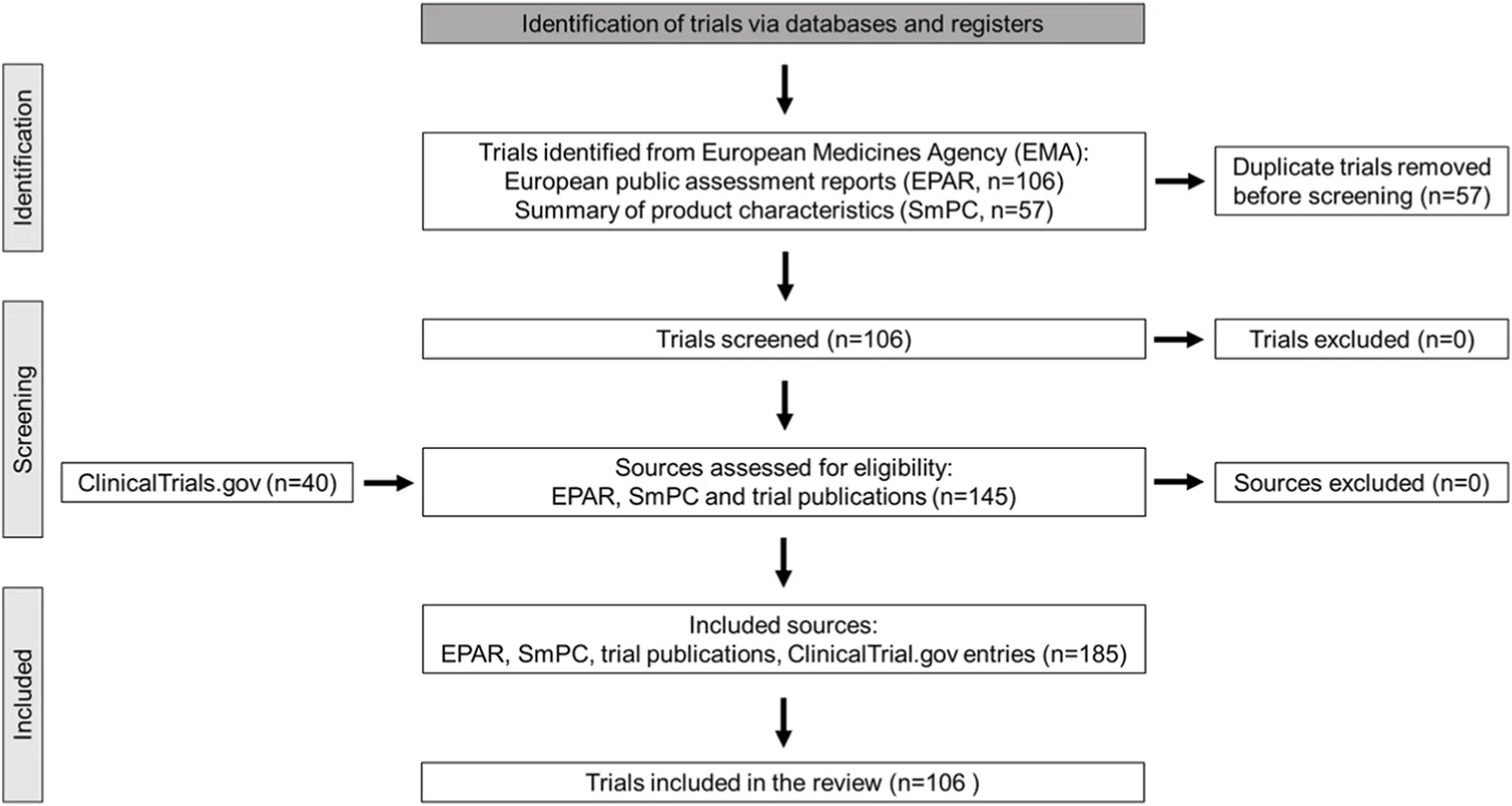
Flowchart of the search process and the sources included

In Tables 1–6, we describe each drug alone with all analyzed trial populations. Each row represents one trial population except for SURMOUNT-OSA (two trial populations in one row). We indicate the individual trials by their acronyms or their respective NCT number from the ClinicalTrials.gov database. In Table 7, a summary for the six drugs is given.

**TABLE 1:** Age-related characteristics of trials with liraglutide.

References	Trial	Age as inclusion criterion	Age of trial population (mean and standard deviation)	Other age-related characteristics (e.g., age range, proportion of age groups)
(European Medicines Agency, 2009; Marre et al., 2009)	LEAD-1 SU	18–80 years	56 (10) years	-
(European Medicines Agency, 2009; Nauck et al., 2009; Nauck et al., 2013; ClinicalTrials.gov. Results Posted, 2024q)	LEAD-2	18–80 years	57 (10) years	-
(ClinicalTrials.gov, 2024; European Medicines Agency, 2009; Garber et al., 2009; Garber et al., 2011; European Medicines Agency, 2016)	LEAD-3 Mono	18–80 years	53 (11) years	≥65 years: 14.5% (n = 108)
(European Medicines Agency, 2009; Zinman et al., 2009)	LEAD-4 Met + TZD	18–80 years	55 (10) years*	-
(European Medicines Agency, 2009; Russell-Jones et al., 2009)	LEAD-5 met + SU	18–80 years	58 (10) years*	-
(European Medicines Agency, 2009; Buse et al., 2009; ClinicalTrials.gov. Results Posted, 2024r)	LEAD-6	18–80 years	57 (10) years	-
(Pratley et al., 2010; ClinicalTrials.gov. Results Posted, 2024s)	1860-LIRA-DPP-4	18–80 years	55 (9) years	-
(ClinicalTrials.gov, 2026a; DeVries et al., 2012; Rosenstock et al., 2013; European Medicines Agency, 2011; European Medicines Agency, 2014a)	NCT00856986	18–80 years	57 (10) years	-
(European Medicines Agency, 2014a; Mathieu et al., 2014; Clinic alTrials.gov, 2026)	BEGIN: VICTOZA ADD-ON	≥18 years	61 (9) years	-
(Zinman et al., 2012; Rodbard et al., 2013; ClinicalTrials.gov, 2025)	BEGIN: Once Long	≥18 years	59 (10) years	>65 years: around 28%
(European Medicines Agency, 2014a; Gough et al., 2014; ClinicalTrials.gov., 2026b)	DUAL I	≥18 years	55 (10) years	-
(European Medicines Agency, 2014a; Buse et al., 2014; ClinicalTrials.gov, 2021)	DUAL II	≥18 years	57 (10) years	-
(Marso et al., 2016a; European Medicines Agency, 2017a; ClinicalTrials.gov. Results Posted, 2024t; European Medicines Agency, 2025b)	LEADER	≥50 years	64 (7) years	≥60 years: 75.1% (n = 7,019) ≥65 years: 46.3% (n = 4,329) ≥75 years: 9% (n = 836) ≥85 years: 0.4% (n = 42)
(European Medicines Agency, 2015; Pi-Sunyer et al., 2015)	SCALE Obesity and Prediabetes	≥18 years	45 (12) years	-
(Davies et al., 2015; European Medicines Agency, 2015; ClinicalTrials.gov. Study Results, 2024d)	SCALE Diabetes	≥18 years	55 (11) years	-
(Blackman et al., 2016; European Medicines Agency, 2015; ClinicalTrials.gov. Results Posted, 2024u)	SCALE Sleep Apnea	18–64 years	49 (10) years	-
(Wadden et al., 2013; European Medicines Agency, 2015; ClinicalTrials.gov. Results Posted, 2024v)	SCALE Maintenance	≥18 years	46 (12) years	-

* Because the publications did not specify the mean age of the overall trial population, we estimated it using the reported mean ages of the treatment and control groups by averaging the means of both arms.

**TABLE 2:** Age-related characteristics of trials with dulaglutide.

References	Trial	Age as inclusion criterion	Age of trial population (mean and standard deviation)	Other age-related characteristics (e.g., age range, proportion of age groups)
(European Medicines Agency, 2014b; Wysham et al., 2014)	AWARD 1	≥18 years	56 (10) years	≥65 years: 16.0% (n = 156) ≥75 years: 1.8% (n = 18)
(European Medicines Agency, 2014b; Giorgino et al., 2015)	AWARD 2	≥18 years	57 (10) years	≥65 years: 20.0% (n = 161) ≥75 years: 1.7% (n = 14)
(European Medicines Agency, 2014b; Umpierrez et al., 2014)	AWARD 3	≥18 years	56 (10) years	≥65 years: 17.7% (n = 143) ≥75 years: 2.6% (n = 21)
(European Medicines Agency, 2014b; Blonde et al., 2015)	AWARD 4	≥18 years	59 (9) years	≥65 years: 27.5% (n = 243) ≥75 years: 3.5% (n = 31)
(European Medicines Agency, 2014b; Weinstock et al., 2015; Nauck et al., 2014)	AWARD 5	18–75 years	54 (10) years	≥65 years: 13.1% (n = 144) ≥75 years: 0.2% (n = 2)
(Dungan et al., 2014; ClinicalTrials.gov. Study Results, 2024e)	AWARD 6	≥18 years	57 (10) years	≥65 years: 18.5% (n = 111)
(Frías et al., 2021a; European Medicines Agency, 2020a)	AWARD 11	≥18 years	57 (10) years	≥65 years: 23.8% (n = 438) ≥75 years: 3.0% (n = 55)
(Gerstein et al., 2019; Gerstein et al., 2018; ClinicalTrials.gov. Results Posted, 2024w; European Medicines Agency, 2019a)	REWIND	≥50 years	66 (7) years	≥65 years: 53.1% (n = 5,256) ≥66 years: 47.1% (n = 4,664)

**TABLE 3:** Age-related characteristics of trials with semaglutide.

References	Trial	Age as inclusion criterion	Age of trial population (mean and standard deviation)	Other age-related characteristics (e.g., age range, proportion of age groups)
(European Medicines Agency, 2022a; Wilding et al., 2021; Wilding et al., 2022)	STEP 1	≥18 years	46 (13) years	≥65 years: 8.0% (n = 156) ≥75 years: 0.6% (n = 11)
(European Medicines Agency, 2022a; Davies et al., 2021)	STEP 2	≥18 years	55 (11) years	≥65 years: 21.6% (n = 174) ≥75 years: 2.2% (n = 18)
(European Medicines Agency, 2022a; Wadden et al., 2021)	STEP 3	≥18 years	46 (13) years	≥65 years: 7.5% (n = 46) ≥75 years: 0.5% (n = 3)
(European Medicines Agency, 2022a; Rubino et al., 2021)	STEP 4	≥18 years	46 (12) years	≥65 years: 6.0% (n = 48) ≥75 years: 0.5% (n = 4)
(Garvey et al., 2022)	STEP 5	≥18 years	47 (11) years	-
(Kadowaki et al., 2022a)	STEP 6	≥18 years	51 (11) years	-
(Rubino et al., 2022)	STEP 8	≥18 years	49 (13) years	-
(Bliddal et al., 2024)	STEP 9	≥18 years	56 (10) years	≥65 years: 18.9% (n = 77) ≥75 years: 3.4% (n = 14) ≥85 years: n = 0
(Kosiborod et al., 2023; ClinicalTrials.gov, 2026c)	STEP-HFpEF	≥18 years	68 (10) years	-
(Kosiborod et al., 2024)	STEP-HFpEF-DM	≥18 years	69 years	-
(European Medicines Agency, 2024a; Lincoff et al., 2023)	SELECT	≥45 years	62 (9) years	≥65 years: 38.2% (n = 6,728) ≥75 years: 7.8% (n = 1,366) ≥85 years: 0.3% (n = 48)
(European Medicines Agency, 2017b; Sorli et al., 2017)	SUSTAIN 1	≥18 years	54 (11) years	age range 18–88 years
(European Medicines Agency, 2017b; Ahrén et al., 2017; ClinicalTrials.gov. Results Posted, 2024x)	SUSTAIN 2	≥18 years	55 (10) years	age range 23–83 years
(European Medicines Agency, 2017b; Rubino et al., 2021; Ahmann et al., 2018; ClinicalTrials.gov. Study Results, 2024f)	SUSTAIN 3	≥18 years	57 (11) years	age range 20–83 years
(European Medicines Agency, 2017b; Aroda et al., 2017; ClinicalTrials.gov. Results Posted, 2024y)	SUSTAIN 4	≥18 years	57 (10) years	age range 22–82 years ≥65 years: 22.4% (n = 242) ≥75 years: 3.9% (n = 42) ≥85 years: n = 0
(European Medicines Agency, 2017b; Rodbard et al., 2018; ClinicalTrials.gov. Results Posted, 2024z)	SUSTAIN 5	≥18 years	59 (10) years	age range 19–86 years ≥65 years: 29.0% (n = 115) ≥85 years: 0.3% (n = 1)
(Marso et al., 2016b; European Medicines Agency, 2017b; E uropean Medicines Agency, 2025)	SUSTAIN 6	≥50 years	65 (7) years	age range 50–89 years ≥65 years: 48.5% (n = 1,598) ≥75 years: 9.7% (n = 320) ≥85 years: 0.6% (n = 19)
(Pratley et al., 2018; ClinicalTrials.gov. Results Posted, 2024c)	SUSTAIN 7	≥18 years	56 (11) years	-
(Zinman et al., 2019a)	SUSTAIN 9	≥18 years	57 (10) years	-
(Kellerer et al., 2022)	SUSTAIN 11	≥18 years	61 (9) years	-
(European Medicines Agency, 2022b; Frías et al., 2021c)	SUSTAIN FORTE	≥18 years	58 (10) years	≥65 years: 28.2% (n = 271) ≥75 years: 3.5% (n = 34)
(European Medicines Agency, 2017b; Seino et al., 2018; ClinicalTrials.gov. Study Details, 2024a)	NCT02254291	≥20 years	58 (11) years	age range 22–83 years
(European Medicines Agency, 2017b; Kaku et al., 2018; ClinicalTrials.gov. Study Details, 2024b)	NCT02207374	≥20 years	59 (10) years	age range 26–83 years
(Perkovic et al., 2024; E uropean Medicines Agency, 2025)	FLOW	≥18 years	67 (9) years	≥65 years: 63.6% (n = 2,248) ≥75 years: 18.7% (n = 659) ≥85 years: 0.8% (n = 30)
(Bonaca et al., 2025; ClinicalTrials.gov., 2026d; European Medicines Agency, 2025)	STRIDE	≥18 years	67 (9) years	<65 years: 36.0% (n = 285) ≥65 years: 64.0% (n = 507)
(European Medicines Agency, 2020b; Aroda et al., 2019)	PIONEER 1	≥18 years	55 (11) years	-
(European Medicines Agency, 2020b; Rodbard et al., 2019)	PIONEER 2	≥18 years	58 (10) years	-
(European Medicines Agency, 2020b; Rosenstock et al., 2019)	PIONEER 3	≥18 years	58 (10) years	-
(European Medicines Agency, 2020b; Pratley et al., 2019)	PIONEER 4	≥18 years	56 (10) years	-
(European Medicines Agency, 2020b; Mosenzon et al., 2019)	PIONEER 5	≥18 years	70 (8) years	-
(Husain et al., 2019; European Medicines Agency, 2020b)	PIONEER 6	≥50 years	66 (7) years	≥65 years: 58.1% (n = 1849)
(European Medicines Agency, 2020b; Pieber et al., 2019)	PIONEER 7	≥18 years	57 (10) years	-
(European Medicines Agency, 2020b; Zinman et al., 2019b)	PIONEER 8	≥18 years	61 (10) years	-
(European Medicines Agency, 2020b; Yamada et al., 2020)	PIONEER 9	≥20 years	59 (9) years	-
(European Medicines Agency, 2020b; Yabe et al., 2020)	PIONEER 10	≥20 years	58 (10) years	≥75 years: 5% (n = 24)
(Aroda et al., 2023)	PIONEER PLUS	≥18 years	58 (11) years	-

**TABLE 4:** Age-related characteristics of trials with tirzepatide

References	Trial	Age as inclusion criterion	Age of trial population (mean and standard deviation)	Other age-related characteristics (e.g., age range, proportion of age groups)
(Rosenstock et al., 2021; European Medicines Agency, 2022c; European Medicines Agency, 2023a)	SURPASS 1	≥18 years	54 (12) years	≥65 years: 22.0% (n = 105) ≥75 years: 2.1% (n = 10) ≥85 years: 0.2% (n = 1)
(European Medicines Agency, 2022c; European Medicines Agency, 2023a; Frías et al., 2021b)	SURPASS 2	≥18 years	57 (10) years	≥65 years: 24.4% (n = 458) ≥75 years: 2.3% (n = 43) ≥85 years: 0.1% (n = 2)
(European Medicines Agency, 2022c; European Medicines Agency, 2023a; Ludv et al., 2021)	SURPASS 3	≥18 years	57 (10) years	≥65 years: 26.4% (n = 379) ≥75 years: 2.9% (n = 42) ≥85 years: n = 0
(European Medicines Agency, 2022c; European Medicines Agency, 2023a; Del Prato et al., 2021)	SURPASS 4	≥18 years	64 (9) years	≥65 years: 47.5% (n = 948) ≥75 years: 9.9% (n = 198) ≥85 years: 0.5% (n = 10)
(European Medicines Agency, 2022c; European Medicines Agency, 2023a; Dahl et al., 2022)	SURPASS 5	≥18 years	61 (10) years	≥65 years: 40.4% (n = 192) ≥75 years: 5.1% (n = 24) ≥85 years: n = 0
(European Medicines Agency, 2022c; Inagaki et al., 2022)	SURPASS-J-mono	≥20 years	57 (10) years	≥65 years: 24.8% (n = 158)
(European Medicines Agency, 2022c; Kadowaki et al., 2022b)	SURPASS-J-combo	≥20 years	57 (11) years	-
(European Medicines Agency, 2023a; Jastreboff et al., 2022)	SURMOUNT 1	≥18 years	45 (13) years	≥65 years: 6% (n = 152) ≥75 years: 0.3% (n = 8)
(Garvey et al., 2023)	SURMOUNT 2	≥18 years	54 (11) years	≥65 years: 18% (n = 165)
(Wadden et al., 2023)	SURMOUNT 3	≥18 years	46 (12) years	-
(European Medicines Agency, 2023a; Aronne et al., 2024)	SURMOUNT 4	≥18 years	48 (13) years	≥65 years: 10.0% (n = 67)
(Malhotra et al., 2024; European Medicines Agency, 2024b)	SURMOUNT-OSA	≥18 years	trial 1: 48 (12) years trial 2: 52 (11) years	trial 1: <50 years: 53.4% (n = 125) ≥50 years: 46.6% (n = 109) trial 2: <50 years: 42.1% (n = 99) ≥50 years: 57.9% (n = 136)

**TABLE 5:** Age-related characteristics of trials with dapagliflozin.

References	Trial	Age as inclusion criterion	Age of trial population (mean and standard deviation)	Other age-related characteristics (e.g., age range, proportion of age groups)
(European Medicines Agency, 2012; Ferrannini et al., 2010; ClinicalTrials.gov. Results Posted, 2024g)	NCT00528372	18–77 years	53 (11) years*	≥65 years 12.7% (n = 71) ≥75 years 0.9% (n = 5)
(ClinicalTrials.gov. Results Posted, 2024d; European Medicines Agency, 2012)	NCT00736879	18–77 years	53 (11) years	≥65 years: 14.9% (n = 42) ≥75 years: 1.4% (n = 4)
(Bailey et al., 2010; European Medicines Agency, 2012; Bailey et al., 2013; ClinicalTrials.gov. Results Posted, 2024h)	NCT00528879	18–77 years	54 (10) years*	age range 29–76 years ≥65 years: 14.5% (n = 79) ≥75 years: 0.7% (n = 4)
(Bolinder et al., 2014; European Medicines Agency, 2012; Ljunggren et al., 2012; ClinicalTrials.gov. Results Posted, 2024i)	NCT00855166	30–75 years	61 (8) years	-
(Wilding et al., 2012; European Medicines Agency, 2012; Wilding et al., 2014; ClinicalTrials.gov. Results Posted, 2024j)	NCT00673231	18–80 years	59 (8) years	≥65 years: 25%** >75 years: <3%**
(Rosenstock et al., 2012; European Medicines Agency, 2012; ClinicalTrials.gov. Results Posted, 2024a)	NCT00683878	≥18 years	54 (11) years	≥65 years: 16% (n = 67) ≥75 years: 2.4% (n = 10)
(Strojek et al., 2011; European Medicines Agency, 2012; ClinicalTrials.gov. Results Posted, 2024k)	NCT00680745	≥18 years	60 (10) years	≥65 years: 32.6%** >75 years: 5.1%**
(Henry et al., 2012; European Medicines Agency, 2012; ClinicalTrials.gov. Results Posted, 2024l)	NCT00643851	18–77 years	52 (10) years	≥65 years: 9.9% (n = 54) ≥75 years: 0.7% (n = 4)
(Henry et al., 2012; European Medicines Agency, 2012; ClinicalTrials.gov. Results Posted, 2024m)	NCT00859898	18–77 years	52 (11) years	≥65 years: 11.8% (n = 75) ≥75 years: 0.8% (n = 5)
(Nauck et al., 2011; European Medicines Agency, 2012; ClinicalTrials.gov. Results Posted, 2024n)	NCT00660907	≥18 years	58 (10) years	age range 24–80 years ≥65 years: 27.5%** ≥75 years: 3.5%**
(ClinicalTrials.gov. Results Posted, 2024e; European Medicines Agency, 2012; Kohan et al., 2014)	NCT00663260	≥18 years	67 (8) years	≥65 years: 58.7% (n = 148)
(ClinicalTrials.gov. Results Posted, 2024f; European Medicines Agency, 2012; Cefalu et al., 2015)	NCT01031680	≥45 years	63 (7) years	≥65 years: 42.5% (n = 388) ≥75 years: 6.5% (n = 59)
(Leiter et al., 2014; European Medicines Agency, 2012)	NCT01042977	≥45 years	64 (7) years*	≥65 years: 47% (n = 451) ≥75 years: 7.7% (n = 75)
(European Medicines Agency, 2019b; Wiviott et al., 2019; European Medicines Agency, 2017c)	DECLARE-TIMI 58	≥40 years	64 (7) years	≥65 years: 46.1% (n = 7,907) ≥75 years: 6.4% (n = 1,096)
(European Medicines Agency, 2020c; McMurray et al., 2019)	DAPA-HF	≥18 years	66 (11) years	>65 years: 57% (n = 2,714) >75 years: 21% (n = 1,003)
(European Medicines Agency, 2022d; Solomon et al., 2022)	DELIVER	≥40 years	72 (10) years	age range 40–99 years >65 years: 76% (n = 4,751) >72 years: 50% (n = 3,114) >75 years: 37.5% (n = 2,347) ≥85 years: 7.2% (n = 450)
(European Medicines Agency, 2021a; Heerspink et al., 2020)	DAPA-CKD	≥18 years	62 (12) years	>65 years: 42% (n = 1818) >75 years: 11% (n = 488)

* Because the publications did not specify the mean age of the overall trial population, we estimated it using the reported mean ages of the treatment and control groups by averaging the means of both arms.

** The publications did not provide the number of participants (n) of each age group.

**TABLE 6:** Age-related characteristics of trials with empagliflozin.

References	Trial	Age as inclusion criterion	Age of trial population (mean and standard deviation)	Other age-related characteristics (e.g., age range, proportion of age groups)
(European Medicines Agency, 2014c; Kovacs et al., 2014; Kovacs et al., 2015)	EMPA-REG PIO EMPA-REG EXTEND PIO	≥18 years	55 (10) years	-
(European Medicines Agency, 2014c; Roden et al., 2013)	EMPA-REG MONO	≥18 years	55 (11) years	-
(European Medicines Agency, 2014c; Häring et al., 2014; Merker et al., 2015)	EMPA-REG MET EMPA-REG EXTEND MET	≥18 years	56 (10) years	-
(European Medicines Agency, 2014c; Häring et al., 2015; Häring et al., 2013)	EMPA-REG METSU EMPA-REG EXTEND METSU	≥18 years	57 (9) years	-
(European Medicines Agency, 2014c; Ridderstråle et al., 2014; ClinicalTrials.gov. Results Posted, 2024o)	EMPA-REG H2H-SU	≥18 years	56 (10) years	-
(European Medicines Agency, 2014c; Rosenstock et al., 2014; ClinicalTrials.gov. Study Details, 2024c)	EMPA-REG MDI	≥18 years	57 (10) years	-
(European Medicines Agency, 2014c; Tikkanen et al., 2015)	EMPA-REG BP	≥18 years	60 (9) years	-
(European Medicines Agency, 2014c; Barnett et al., 2014; ClinicalTrials.gov. Results Posted, 2024b)	EMPA-REG RENAL	≥18 years	64 (9) years	-
(European Medicines Agency, 2014c; European Medicines Agency, 2017d; Zinman et al., 2015)	EMPA-REG OUTCOME	≥18 years	63 (9) years	≥65 years: 44.5% (n = 3,127) ≥75 years: 9.3% (n = 652)
(European Medicines Agency, 2021b; European Medicines Agency, 2022e; Packer et al., 2020)	EMPEROR-reduced	≥18 years	67 (11) years	≥65 years: 62.1% (n = 2,315) ≥75 years: 26.8% (n = 999) ≥85 years: 2.7% (n = 99)
(Anker et al., 2021; European Medicines Agency, 2022e; European Medicines Agency, 2025e)	EMPEROR-preserved	≥18 years	72 (9) years	≥70 years: 64.1% (n = 3,838)
(Abraham et al., 2021; European Medicines Agency, 2022e)	EMPERIAL-reduced	≥18 years	69 (10) years	≥65 years: 69.8% (n = 217)
(Abraham et al., 2021; European Medicines Agency, 2022e)	EMPERIAL-preserved	≥18 years	74 (9) years	≥65 years: 85.7% (n = 270)
(European Medicines Agency, 2022e; Hundertmark et al., 2023)	EMPA-VISION	≥18 years	68 (12) years	-
(Herrington et al., 2023; European Medicines Agency, 2023b; European Medicines Agency, 2025e; ClinicalTrials.gov. Results Posted, 2024p)	EMPA-KIDNEY	≥18 years	63 (14) years	≥75 years: 23% (n = 1,518) ≥85 years: 2% (n = 133)

**TABLE 7:** Summary of age proportion and trials published age-stratified outcomes.

Drug	Indications/outcome	Trials reporting age ≥65 years	Patient proportion	Trials reporting age ≥75 years	Patient proportion	Trials reporting age ≥85 years	Patient proportion	Trials with age-stratified efficacy outcomes*	Trials with age-stratified safety outcomes*
			Median (range)		Median (range)		Median (range)		
Liraglutide	All (n = 17)	12% (n = 2)	30.4% (14.5%–46.3%)	6% (n = 1)	9.0%	6% (n = 1)	0.4%	6% (n = 1)	12% (n = 2)
	Glycemic control (n = 12)	8% (n = 1)	14.5%	0	-	0	-	0	8% (n = 1)
	Weight management (n = 4)	0	-	0	-	0	-	0	0
	CVOT (n = 1)	100% (n = 1)	46.3%	100% (n = 1)	9.0%	100% (n = 1)	0.4%	100% (n = 1)	100% (n = 1)
Dulaglutide	All (n = 8)	100% (n = 8)	19.3% (13.1%–53.1%)	75% (n = 6)	2.2% (0.2%–3.5%)	0	-	13% (n = 1)	13% (n = 1)
	Glycemic control (n = 7)	100% (n = 7)	18.5% (13.1%–27.5%)	86% (n = 6)	2.2% (0.2%–3.5%)	0	-	0	14% (n = 1)
	Weight management (n = 0)	-	-	-	-	-	-	-	-
	CVOT (n = 1)	100% (n = 1)	53.1%	0	-	0	-	100% (n = 1)	0
Semaglutide	All (n = 36)	36% (n = 13)	28.2% (6.0%‐64.0%)	31% (n = 11)	3.5% (0.5%–18.7%)	17% (n = 6)	0.3% (0%–0.8%)	14% (n = 10)	6% (n = 2)
	Glycemic control (n = 21)	14% (n = 3)	28.2% (22.4%–29.0%)	14% (n = 3)	3.9% (3.5%–5.0%)	10% (n = 2)	0.2% (0%–0.3%)	5% (n = 1)	5% (n = 1)
	Weight management (n = 8)	60% (n = 5)	8.0% (6.0%–21.6%)	60% (n = 5)	0.6% (0.5%–3.4%)	13% (n = 1)	0	50% (n = 4)	0
	CVOT, HF, CV risk factors, pAD, CKD (n = 7)	71% (n = 5)	58.1% (38.2%–64.0%)	43% (n = 3)	9.7% (7.8%–18.7%)	43% (n = 3)	0.6% (0.3%–0.8%)	71% (n = 5)	14% (n = 1)
Tirzepatide	All (n = 13)	70% (n = 9)	24.4% (6.0%–47.5%)	46% (n = 6)	2.6% (0.3%–9.9%)	38% (n = 5)	0.1% (0%–0.5%)	46% (n = 6)	0
	Glycemic control (n = 7)	86% (n = 6)	25.6% (22.0%–47.5%)	71% (n = 5)	2.9% (2.1%–9.9%)	71% (n = 5)	0.1% (0%–0.5%)	71% (n = 5)	0
	Weight management (n = 4)	75% (n = 3)	10.0% (6.0%–18.0%)	25% (n = 1)	0.3%	0	-	25% (n = 1)	0
	OSA (n = 2)	0	-	0	-	0	-	0	0
Dapagliflozin	All (n = 17)	76% (n = 13)	25.0% (9.9%–58.7%)	59% (n = 10)	1.9% (0.7%–7.7%)	6% (n = 1)	7.2%	35% (n = 6)	35% (n = 6)
	Glycemic control (n = 10)	100% (n = 10)	15.5% (9.9%–58.7%)	70% (n = 7)	0.9% (0.7%–3.5%)	0	-	0	0
	Weight management (n = 0)	-	-	-	-	-	-	-	-
	CVD, HF, CKD, bone density, body weight, blood pressure (n = 7)	43% (n = 3)	46.1% (42.5%–47.0%)	43% (n = 3)	6.5% (6.4%–7.7%)	14% (n = 1)	7.2%	86% (n = 6)	86% (n = 6)
Empagliflozin	All (n = 15)	27% (n = 4)	66.0% (44.5%–85.7%)	20% (n = 3)	23.0% (9.3%–26.8%)	13% (n = 2)	2.4% (2.0%–2.7%)	40% (n = 6)	13% (n = 2)
	Glycemic control (n = 5)	0	-	0	-	0	-	0	0
	Weight management (n = 0)	-	-	-	-	-	-	-	-
	CVOT, HF, blood pressure, obesity, CKD (n = 10)	40% (n = 4)	66.0% (44.5%–85.7%)	30% (n = 3)	23.0% (9.3%–26.8%)	20% (n = 2)	2.4% (2.0%–2.7%)	60% (n = 6)	20% (n = 2)
All	All (n = 106)	46% (n = 49)	-	35% (n = 37)	-	14% (n = 15)	-	28% (n = 30)	12% (n = 13)

CVOT, cardiovascular outcome trial; CV, cardiovascular; CVD, cardiovascular disease; HF, heart failure; OSA, obstructive sleep apnea; pAD, peripheral arterial disease; CKD, chronic kidney disease.

* pooled data of several trials published in EPAR are not included.

### 3.2 Liraglutide

The publications of the examined trials and the corresponding EPAR (Table 1) provide only limited information on the proportion of older participants or on the efficacy and safety of liraglutide in this subgroup. In our search, the proportion of older participants could be determined for only three trials (ClinicalTrials.gov, 2024; Zinman et al., 2012; Marso et al., 2016a). Some trials limited the inclusion age of participants to a maximum age of 80 or even 64 years (Table 1).

The cardiovascular outcome trial LEADER included patients with a minimum age of 50 years (Marso et al., 2016a). The publication reports the hazard ratios for the primary composite outcome - defined as first occurrence of cardiovascular death, non-fatal (including silent) myocardial infarction, or non-fatal stroke - for two subgroups: participants younger than 60 years and those aged 60 years or older. In the older age group, liraglutide tended to show a benefit compared to placebo, although this was not statistically significant.

Some EPAR (pooled data from the LEAD 1–5 trials) state that reductions in HbA1c were comparable across age groups (<60 years; >60 years; >70 years; and ≥75 years), but they do not provide the underlying numerical data (European Medicines Agency, 2009). The safety analysis shows higher rates of adverse events, serious adverse events and discontinuation due to adverse events with increasing age in the liraglutide group, mainly for gastrointestinal symptoms. Another EPAR shows a forest plot analysis of the reduction in HbA1c levels after 3 years in different age groups compared to placebo, based on the LEADER trial (European Medicines Agency, 2017a). The effect between the groups was consistent and with a clear advantage for liraglutide, but not significant in the ≥85-year-old group due to the wide confidence interval. The safety analysis showed an increase in adverse events and serious adverse events with increasing age, but this was significantly different between the placebo and liraglutide group (European Medicines Agency, 2017a).

The publications of the SCALE trials do not include age subgroup analyses (Pi-Sunyer et al., 2015; Davies et al., 2015; Blackman et al., 2016; Wadden et al., 2013). The related EPAR shows the proportions of participants in different age groups, pooled data from the SCALE trials and a phase 2 trial result in a proportion of 6.6% (n = 384) with an age of at least 65 years and 0.4% (n = 22) of at least 75 years (European Medicines Agency, 2015). Accordingly, the assessment of safety in these age groups is also limited. For example, 41% of 75–84 years old patients discontinued trial medication due to adverse events. Given the small number of older participants in the clinical trials, the EMA states that therapeutic experience with liraglutide in patients aged 75 years and older is insufficient, and its use in this population cannot be recommended (European Medicines Agency, 2015; European Medicines Agency, 2025a).

### 3.3 Dulaglutide

The proportion of patients aged 65 years and older is stated for all dulaglutide trials and ranged between 13.1% and 53.1% (Table 2). However, the proportion of patients ≥75 years, which was additionally published for some trials, was only 0.2%–3.5% and the highest absolute number in a trial was n = 55 patients. Taken together, the trial populations of the AWARD 1-5 trials included 18.5% (n = 847) patients aged ≥65 years and 1.9% (n = 86) aged ≥75 years (European Medicines Agency, 2014b). Only the AWARD 5 trial limited the inclusion age to a maximum of 75 years (Weinstock et al., 2015). None of the publications from the AWARD 1–6, 11, or REWIND trials included analyses stratified by age group.

The cardiovascular outcome trial REWIND included patients aged 50 years or older, the total number of participants was significantly higher than in AWARD 1-6 or AWARD 11 and 47.1% were aged 66 years or older (Gerstein et al., 2019). The subgroup analysis of the incidence of the primary composite outcome (first occurrence of non-fatal myocardial infarction, non-fatal stroke or cardiovascular-related death including unknown cause of death) among patients up to 65 years and 66 years and older was not significantly different between dulaglutide and placebo groups and similar in the age groups.

The EPAR shows results of a pharmacokinetic trial with no significant differences in the age groups ≥65 years and ≥75 years for the area under the curve (AUC) and the maximum plasma concentrations (c_max_) (European Medicines Agency, 2014b). The EPAR also includes aggregated data from several placebo-controlled trials, which showed no age-related differences regarding safety. When using 1.5 mg dulaglutide, adverse events occurred more frequently in the age group ≥75 years compared to 0.75 mg, especially gastrointestinal disorders, nervous system disorders (e.g., headache) and decreased appetite, but in each case without significant difference.

The SmPC states that age has no clinically meaningful impact on the pharmacokinetic or pharmacodynamic profile (European Medicines Agency, 2025c).

### 3.4 Semaglutide

The inclusion criteria of the examined trials with semaglutide did not specify a maximum age (Table 3). For some trials (e.g., SUSTAIN 1-6), information on the range of participants age could be obtained. Around 30% of the trials and publications indicated proportions of different age groups in the trial population, ranging from 6.0%–64.0% for patients aged 65 years or older and from 0.5%–18.7% for patients aged 75 years or older. Age-related subgroup analyses have been published for the cardiovascular outcome trials SELECT, SUSTAIN 6, and PIONEER 6.

One of the EPAR contains information on defined age groups (European Medicines Agency, 2022a). These data show that a total of 10.1% (n = 424) of the participants in the STEP 1-4 trials were 65 years or older, but only 0.9% (n = 36) were at least 75 years old. The mean plasma concentration of semaglutide (STEP 1 trial population) in the age groups 65–74 years and ≥75 years compared to 18–64 years is shown in the report as a forest plot. No significantly different values were observed in the groups. Forest plot analyses of the placebo-controlled STEP 1-4 trials of the percentage reduction in body weight with semaglutide show a significant benefit of semaglutide for both, those under 65 years of age and those ≥65 years of age, the latter with greater variability. Adverse events, in particular gastrointestinal disorders, occurred more frequently in the ≥65 years age group, but no specific figures are given.

The SELECT trial, which investigated cardiovascular outcomes in patients with obesity (without diabetes mellitus), included participants aged 45 years or older. The publication shows the proportion of patients in different age groups (Lincoff et al., 2023). For example, 7.8% of patients were at least 75 years old, the absolute number (n = 1,366) is obviously larger compared to other trials (with available data). A forest plot analysis of the primary composite outcome (time to first occurrence of cardiovascular-related death, non-fatal myocardial infarction or non-fatal stroke) shows the hazard ratio for the age groups of <55 years, 55 to <65 years, 65 to <75 years and 75 years and older. For all groups, semaglutide tended to have an advantage compared to placebo; however, this was only significant in the 55 to <65 years and 65 to <75 years groups, with the smallest trend and greatest variability in the ≥75-year-olds. EMA points out that the therapeutic experience in patients aged at least 85 years is limited (European Medicines Agency, 2024a).

In the SUSTAIN 6 cardiovascular outcome trial, a minimum age of 50 years was an inclusion criterion; a forest plot subgroup analysis in the supplementary appendix of published data showed no significant difference between the two age groups for participants under 65 years of age and those aged 65 years or older with regard to the primary composite outcome (first occurrence of cardiovascular-related death, non-fatal myocardial infarction or non-fatal stroke) and a trend towards an advantage of semaglutide in each case, although this was not significant (Marso et al., 2016b).

Another EPAR also contains information on the average plasma concentration of semaglutide in various subgroups, there is no significant difference between the age groups 65–74 years and >74 years and 18–64 years (European Medicines Agency, 2017b). According to this EPAR, the reduction of the HbA1c level by semaglutide was consistent in all age groups, in some trials slightly lower in the over 75-year-old patients, but due to the small number of participants in this age group an evaluation of efficacy was not possible. The pooled data from several phase 3a trials (SUSTAIN 1–5, NCT02254291, NCT02207374) also report adverse event rates stratified by age group. Overall, the safety profile was assessed as consistent, but the group of ≥75-year old patients (n = 156) was significantly smaller than the younger age groups (n = 4,651). The EPAR displays the change in HbA1c using 2.0 mg or 1.0 mg semaglutide in the SUSTAIN FORTE trial and a subgroup analysis for the age groups under 65 years, 65–74 years and over 75 years. Only the group under 65 years of age showed a significant advantage for 2.0 mg semaglutide. The occurrence of adverse events in these age groups was slightly more frequent in the group of ≥75-year-old patients (n = 34) with 1.0 mg semaglutide.

The highest proportion of patients aged 65 years or older is represented in the FLOW and STRIDE trials and is around 64%. The publication of the FLOW trial shows the primary composite outcome, the occurrence of major kidney disease events, in subgroups aged younger than 65 years, between 65 and 74 years and at least 75 years with beneficial effects of semaglutide compared to placebo in all age groups, significantly in patients aged <65 years and ≥75 years (Perkovic et al., 2024). The placebo-controlled STRIDE trial included patients with type 2 diabetes mellitus and peripheral artery disease (Bonaca et al., 2025). The ratio to baseline of the maximum walking distance at week 52 as the primary outcome favors semaglutide in both age groups, <65 and ≥65 years.

The age of inclusion in the PIONEER 6 trial was at least 50 years, the proportion of ≥65-year-old patients was 58.1% (Husain et al., 2019). Subgroup analyses of the primary composite outcome (first occurrence of cardiovascular-related death including undetermined causes of death, non-fatal myocardial infarction or non-fatal stroke) showed a significant difference between the age groups. There was an advantage for semaglutide among patients aged 50–64 years; but no significant difference compared to placebo in patients aged 65 years and older.

The age of 39% (n = 3,749) of all patients in the PIONEER 1-10 trials was 65 years or older according to the EPAR, but only 7.7% (n = 730) were at least 75 years old and around 0.3% (n = 24) at least 85 years (European Medicines Agency, 2020b). No age dependency was mentioned for blood glucose-lowering activity, but data were not presented. A pooled analysis of adverse events in the PIONEER 1-5 and 7-10 trials shows an increasing frequency of discontinuation of semaglutide with age. In particular, gastrointestinal disorders, reduced appetite, weight loss and falls were recorded more frequently in age groups ≥65 years and older. According to the report, causing falls (7 vs. 1 patient) by semaglutide cannot be ruled out. In general, the EMA points out that therapeutic experience is limited in patients aged ≥75 years (European Medicines Agency, 2020b; European Medicines Agency, 2025d).

### 3.5 Tirzepatide

No trial listed in Table 4 had an upper age limit. Most publications indicate the proportion of older patients in the trial population. If data are available, between 6%–47.5% were ≥65 years and 0.3%–9.9% were ≥75 years old.

A subgroup analysis of the reduction in HbA1c levels after 40 weeks for tirzepatide 5 mg, 10 mg and 15 mg compared to placebo has been published for the SURPASS 1 trial (Rosenstock et al., 2021). Tirzepatide was significantly beneficial in both age groups, with comparatively greater effect in patients aged <65 years.

A meta-analysis including cardiovascular outcomes from the data of a phase 2 trial as well as SURPASS 1-5 and SURPASS J-mono presents the hazard ratio of MACE-4 (cardiovascular-related death, myocardial infarction, stroke or hospitalized unstable angina) compared to placebo for participants up to 64 years and 65 years and older (31.9%, n = 2,298) (Sattar et al., 2022). The data showed no significant difference between the age groups as well as no significant benefit of tirzepatide, which tended to be shown for the group aged 65 years and older.

One of the EPAR shows the respective number of participants in different age groups of 19 phase 1-3 trials (European Medicines Agency, 2022c). Overall, around 25% (n = 1,427) were between 65 and 74 years old and 4% (n = 214) were at least 75 years old. In the phase three trials SURPASS 1-5, 5.1% (n = 317) were at least 75 years old and 0.2% (n = 13) ≥ 85 years old. For each of the SURPASS 1-5 trials a forest plot subgroup analysis of reduction in HbA1c levels after 40 weeks for tirzepatide 5 mg, 10 mg and 15 mg in the age groups under 65 years and 65 years and older and additionally under 75 years and 75 years and older with data from SURPASS 2-5 is shown. The data mostly showed a benefit for tirzepatide. There was no significant difference in patients aged ≥75 years in SURPASS 2 and 3 trials for all doses and in SURPASS 4 for 10 mg, as well as in SURPASS 2 in ≥65-year-olds with 5 mg. The reduction in HbA1c levels with tirzepatide was slightly more pronounced in the younger age groups.

Pooled safety data from phase 2 and 3 trials are presented in the EPAR for subgroups aged under 65 years, 65–74 years, 75–84 years and 85 years and older. Accordingly, in SURPASS 1-5, SURPASS J-mono and SURPASS J-combo trials, serious adverse events, adverse events leading to discontinuation of trial medication, accidents and injuries, nervous system disorders, cardiac and vascular disorders, hypotension, falls and fractures occurred more frequently with increasing age in both, the tirzepatide and the comparator group. The proportion of participants with gastrointestinal adverse events (especially diarrhea and vomiting) and the resulting discontinuation of trial medication in the tirzepatide group increased with age. Due to the small number of patients aged ≥85 years, EMA stated that the tolerability for this age group could not be assessed (European Medicines Agency, 2022c).

A further EPAR describes the proportions of ≥65 and ≥75-year-old patients in the trial population for SURMOUNT 1 and 4 and the group of patients with overweight or obesity from SURPASS 1-5 (European Medicines Agency, 2023a). The proportion of patients aged 75 years or older was between 0.3% and 9.0% (total n = 261 ≥ 75 years). The forest plot analysis with data from the SURMOUNT 1 trial shows the change in body weight for participants below 65 years and older compared to placebo; tirzepatide showed a significant benefit for all dosages (5 mg, 10 mg, 15 mg), with no significant difference between the age groups. A pooled analysis of the change in body weight of patients aged ≥75 years in the SURMOUNT 1 and SURPASS 1-5 trials showed a significant reduction compared to baseline weight and also placebo. However, the EMA noticed a limited availability of data for patients aged 85 years and older (European Medicines Agency, 2023a).

### 3.6 Dapagliflozin

Except for a single trial, the publications we examined provided sufficient information to determine the proportion of older participants (Table 5). The publications related to type 2 diabetes do not present any subgroup analyses based on individual age categories (Ferrannini et al., 2010; ClinicalTrials.gov. Results Posted, 2024d; Bailey et al., 2010; Bolinder et al., 2014; Wilding et al., 2012; Rosenstock et al., 2012; Strojek et al., 2011; Henry et al., 2012; Nauck et al., 2011). Some of these trials imposed upper age limits of 77, 75, or 80 years, and the proportion of participants aged ≥65 years did not exceed one third. If proportions of ≥75-year-olds were specified, these were between 0.7% and 5.1%. The absolute number of patients aged at least 65 as well as 75 years was significantly higher in cardiovascular and/or renal outcome trials, with between 42% and 76% of the trial population aged 65 years or older (ClinicalTrials.gov. Results Posted, 2024e; ClinicalTrials.gov. Results Posted, 2024f; Leiter et al., 2014; Wiviott et al., 2019; McMurray et al., 2019; Solomon et al., 2022; Heerspink et al., 2020).

According to one EPAR, the pharmacokinetics of dapagliflozin do not differ in individuals aged up to 70 years (European Medicines Agency, 2012; European Medicines Agency, 2024c). For older age, a possibly increased exposure due to reduced renal function is expected, but could not conclusively be assessed due to insufficient data. The report also analyzed pooled data from all phase 3 trials included (the first eleven trials in Table 5) and reported a proportion of 21.3% (n = 1,212) of participants aged ≥65 years and 2.8% (n = 157) aged ≥75 years. An analysis of the reduction in HbA1c after 24 weeks showed significant benefits of dapagliflozin compared to placebo for <65 years and 65 years and older, but no significance in the group aged 75 years and older. The analysis of the safety data in the EPAR is based on three phase 2 trials and eleven phase 3 trials. Renal insufficiency or renal failure and hypovolemia-related events occurred slightly more frequently in the subgroup aged ≥65 years. Overall, (serious) adverse events were not reported significantly more frequently in older patients than in the overall population. However, the EMA points out that the data on ≥ 75-year-olds are limited and that the risk of volume deficiency and renal insufficiency is increased in this patient group.

Three trials with metabolic, cardiovascular or associated risk-factor outcomes (DECLARE, NCT01031680, NCT01042977) included patients aged 40, 45 and 50 years and older (Leiter et al., 2014; Wiviott et al., 2019; Cefalu et al., 2015). The proportion of at least 65-year-olds is over 40%, those aged 75 years and older, if specified, only reaches the single-digit percentage range. All three trials include an analysis for the age group <65 years and 65 years and older, the latter trial (NCT01042977) additionally for ≥75 years. In the DECLARE trial, there was no significant difference between the age groups for the combined outcome of cardiovascular-related death and hospitalization for heart failure, but a significant benefit of dapagliflozin only for those aged ≥65 years (Wiviott et al., 2019). The occurrence of MACE (major adverse cardiovascular events; cardiovascular-related death, myocardial infarction, ischemic stroke) was comparable between the age groups and between placebo and dapagliflozin. The corresponding EPAR summarizes the adverse events for the age group of 75 years and older; the occurrence of events in the dapagliflozin and placebo groups was balanced (European Medicines Agency, 2019b). For example, there was a slightly higher incidence of fractures and urinary tract infections in the dapagliflozin group, but renal events in the placebo group. Overall, the occurrence of adverse events was more frequent with increasing age, but the safety profile of the older trial participants was judged in the report as comparable to the overall population.

The publications of the two other trials (NCT01031680, NCT01042977) present the results for the efficacy of dapagliflozin, for example, the change in HbA1c, body weight or systolic blood pressure, and showed a statistically significant difference compared to placebo in both age groups, with the exception of the change in systolic blood pressure (Leiter et al., 2014; Cefalu et al., 2015). The safety data are also presented in detail; the occurrence of adverse events was consistent and similar between placebo and dapagliflozin in both trials for all age groups. The proportion of participants who discontinued the trial due to adverse events increased with age in both groups. Renal insufficiency or renal failure in both trials occurred more frequently with increasing age as well as in the dapagliflozin group.

In the pivotal trials for chronic heart failure and chronic kidney disease, older patients were more prominently represented (McMurray et al., 2019; Solomon et al., 2022; Heerspink et al., 2020). The proportion of patients in the respective age groups is balanced. A total of 9,283 people aged >65 years and 3,838 people >75 years were included in the three trials. The age of inclusion in the DELIVER trial was ≥40 years. In the DELIVER trial, eligibility required a minimum age of 40 years. The publications of all three trials (DELIVER, DAPA-HF, DAPA-CKD) present the hazard ratio of the primary outcome for the age groups ≤65 or ≤72 years and >65 or >72 years and show a comparable and significant benefit of dapagliflozin compared to placebo for all groups.

The EPAR lists safety data from the DAPA-HF trial regarding the occurrence of volume depletion, renal events and fractures, the incidence was comparable between placebo and dapagliflozin, with a slight increase in the 65+ age group (European Medicines Agency, 2020c). Adverse events, particularly serious adverse events, which occurred in the DAPA-CKD trial were slightly increased in the age group 65 years and older according to the EPAR (European Medicines Agency, 2021a). The primary outcome of the DELIVER trial was analyzed in the corresponding EPAR for the different age groups less than 65 years, 65–74 years, 75–84 years and older and showed no significant difference between the groups (European Medicines Agency, 2022d). Adverse events occurred more frequently in the age groups >65 and >75 years and trial medication was discontinued slightly more frequently due to adverse events with higher age under dapagliflozin. Taken together, the EMA concluded that the safety findings from the three trials were consistent among the different age groups (European Medicines Agency, 2020c; European Medicines Agency, 2021a; European Medicines Agency, 2022d).

### 3.7 Empagliflozin

The proportions of the different age groups were only given for the cardiovascular outcome trials and trials in chronic heart failure and chronic kidney disease patients (except EMPA-VISION) (Zinman et al., 2015; Packer et al., 2020; Anker et al., 2021; Abraham et al., 2021; Herrington et al., 2023). In these trials, there is a high representation of ≥65-year-olds. None of the trials listed in Table 6 specified a maximum age for inclusion.

According to the EPAR, the therapeutic effect (change in HbA1c) is lower in patients aged 75 years and older compared to younger patients and also insufficient, at least with a GFR of 45–60 mL/min (European Medicines Agency, 2014c). With a GFR of more than 60 mL/min, the effectiveness is judged by the EMA as possibly sufficient in over 75-year-olds. The EPAR also contains a pooled analysis of all trials with patients with type 2 diabetes mellitus which included 4,164 participants aged 65 years or older (around 32%), but only 760 were aged at least 75 years. The associated safety data show a more frequent occurrence of adverse events with increasing age for the intervention and control groups. Volume depletion, reduced renal function or urinary tract infections occurred slightly more frequently in the empagliflozin group with increasing age, but serious adverse events occurred less frequently in the empagliflozin group than in the control group.

The publication of the EMPA-REG OUTCOME trial includes a subgroup analysis of the hazard ratio of the primary composite outcome (cardiovascular-related death, non-fatal myocardial infarction, non-fatal stroke) and the occurrence of cardiovascular-related death (Zinman et al., 2015). While in the age group under 65 years no significant efficacy was detected, the participants aged 65 years and older showed a benefit with empagliflozin compared to placebo. Inclusion in the trial was possible from the age of at least 18 years, but the proportion of patients aged <50 years was 6.3% according to the EPAR, 35.3% were aged between 65 and 74 years and 9.3% of participants were older than 74 years (European Medicines Agency, 2017d). The report also shows a forest plot analysis of the primary outcome (hazard ratio) for 10 mg and 25 mg empagliflozin compared to placebo in the age groups under 50 years, 50–64 years, 65–74 years and 75 years and older. Except for the age group 65–74 years with 10 mg empagliflozin, the results did not show a significant benefit of empagliflozin in any group. The safety data indicate an increasing frequency of (serious) adverse events with age and discontinuation of trial medication due to adverse events, both in the empagliflozin and placebo groups.

The proportion of participants of different age groups in the EMPEROR-reduced trial is shown in the corresponding EPAR; for example, 26.8% of the trial population was aged at least 75 years (European Medicines Agency, 2021b). The publication of the trial shows in a forest plot a benefit for empagliflozin compared to placebo for participants aged <65 years and >65 years with regard to the primary composite outcome (cardiovascular death or hospitalization due to heart failure) (Packer et al., 2020). In the EMPEROR-preserved trial, this analysis showed a significant benefit of empagliflozin in the age group 70 years and older, but not younger than 70 years (Anker et al., 2021). The same conclusion was reached for hospitalization due to heart failure as a single outcome, which is presented in the corresponding EPAR (European Medicines Agency, 2017d). The analysis of the decrease in estimated glomerular filtration rate (eGFR) as a further outcome showed a benefit for the empagliflozin group for both age groups. The report also analyzed these outcomes for the pooled data of the EMPEROR-reduced and EMPEROR-preserved trials, which showed no significant difference between empagliflozin and placebo for the age group under 50 years. The same applies to the 85+ age group, except for the reduction in eGFR. With regard to cardiovascular death as an outcome, there was no difference in any age group. In all other outcomes, there was a benefit for empagliflozin in 50–64 years, 65–74 years and 75–84 years. Based on the data from both EMPEROR trials, the EMA assessed the efficacy and benefits for ≥85-year-olds with chronic heart failure as apparently equivalent or no less than for younger age groups.

The safety analysis of the pooled data from the EMPEROR-reduced, -preserved, EMPERIAL-reduced, -preserved and EMPA-VISION trials showed a slight increase in (serious) adverse events, or discontinuation of trial medication due to adverse events with increasing age in the empagliflozin and placebo groups.

A subgroup analysis of the EMPERIAL-reduced and -preserved trials compared patients younger and older than 65 years with regard to the primary outcome “changes in the 6-min walk test after 12 weeks” (Abraham et al., 2021). Empagliflozin was not superior to the placebo group in any age group.

For the publication of the EMPA-KIDNEY trial, the data on the primary composite outcome (progression of kidney disease or cardiovascular death) are presented for the age groups under 60 years, between 60 and 69 years and 70 years and older, with a benefit for empagliflozin compared to placebo in all groups, although not significant in the 60–69 years-age group (Herrington et al., 2023). The proportion of participants aged 70 years and older was 40%. The EPAR shows the evaluation of the primary outcome for a different allocation of age groups, resulting in no significant effect for the group of participants under 50 years of age and those older, although the latter group comprised a comparatively small number of patients (European Medicines Agency, 2023b). Adverse events occurred more frequently in both, the placebo and empagliflozin groups, with increasing age.

### 3.8 Summary

The analysis of 106 trials of the GLP1-RA liraglutide, dulaglutide, semaglutide, tirzepatide, and SGLT2-I dapagliflozin and empagliflozin shows inconsistent reporting of age-stratified data such as age proportions or efficacy and safety outcomes. Table 7 summarizes the data for all drugs (based on Tables 1–6) to provide an overview about the proportion and reporting of outcomes for different age groups. In most cases, the categories ≥65 years, ≥75 years, and ≥85 years were used in the sources to describe the trial populations. In Table 7, the number of trials reporting the proportion of patients aged ≥65 years, ≥75 years, and ≥85 years for each drug is shown, as well as summarized data for all drugs. Moreover, the median and range of the proportion of patients in this age groups are provided. For easier reading, the few trials with other age categorizations are not included in Table 7. As the trials focused on different trial populations, indications, or outcomes such as glycemic control or weight management as an outcome, or patients with cardiorenal comorbidities, Table 7 contains a differentiation of these characteristics. The included trials and sources rarely reported age-stratified efficacy and safety outcomes. If data are provided, they usually show the primary outcomes (e.g., change in glycated hemoglobin) for different age-groups. In general, safety outcomes are mentioned as pooled data in the EPAR (not included in Table 7).

## 4 Conclusion

This review aimed to analyze if older patients were represented in the populations of GLP1-RA and SGLT2-I pivotal trials considered for approval by EMA. Nearly one half of the analyzed sources did not comprise any information about age composition, not to mention outcomes in different age groups. The finding demonstrates a significant lack of reporting of age-related trial results. Furthermore, where age-specific data are available, older patients are underrepresented in most trials, especially patients aged 75 years or older. Comparing these trials, we found a higher proportion of older patients in trials with cardiovascular or renal outcomes. Further, patients with typical geriatric syndromes such as frailty - which may be an important key population based on the indications of these drugs - were not assessed individually as a subgroup.

We also searched for age-specific reports on outcome within the included pivotal trials. Those data were rather rare. With respect to clinical efficacy, the picture is not consistent, sometimes older patients had similar outcomes than younger patients, sometimes older patients did not profit from the intervention, even when the outcome in the total trial population was positive. If safety results for different age groups were reported, they often show a higher frequency of ADR with increasing age, but mostly in both, the intervention and control groups. Overall, the available data from the trials included in this review do not allow to draw clinical relevant age-related conclusions with respect to safety and tolerability.

The detected incomplete reporting of age-related data also leads to varying EMA statements as summarized in the respective EPAR. A summary of relevant findings and statements of the EMA within the EPAR or SmPC is shown in Table 8. Presented are results or statements which indicate a difference between age groups. Differences shown in Table 8 refer exclusively to the intervention group. If Table 8 reports no evidence of differences (“no”), this does not mean that differences do not exist, as data gaps are common. More detailed information about differences identified or the overall results of subgroup analyses are already described in the respective sections on each drug. Overall, the EMA statements clearly point to the limited evidence for prescribing the analyzed drugs in older patients obtained in the respective pivotal trials.

**TABLE 8:** EMA statements on efficacy and safety in the EPAR and SmPC regarding differences between age groups.

​	Is there evidence for clinically relevant differences between age groups?				EPAR/SmPC
Drug	Efficacy		Safety		References (manuscript)
Liraglutide	No/yes*	The EMA states that the use of one of the two products available on the market (indication: weight management) is not recommended in this population due to insufficient therapeutic experience in patients aged ≥75 years	yes	Based on some trials, there is a higher frequency of AE, SAE and discontinuation of trial medication due to AE with increasing age, especially gastrointestinal symptoms The EMA states that the use of one of the two products available on the market (indication: weight management) is not recommended in this population due to insufficient therapeutic experience in patients aged ≥75 years	(European Medicines Agency, 2009; European Medicines Agency, 2017a; European Medicines Agency, 2015; European Medicines Agency, 2025a; European Medicines Agency, 2025b)
Dulaglutide	No	-	no	-	(European Medicines Agency, 2014b; European Medicines Agency, 2025c)
Semaglutide	No/yes*	The EMA states that the therapeutic experience in patients aged ≥85 years (indication: weight management) or ≥75 years (oral product; indication: diabetes mellitus type 2) is limited	no/yes*	Based on some trials, a higher frequency of AE (and discontinuation of trial medication due to AE) with increasing age is described, especially gastrointestinal symptoms	(European Medicines Agency, 2022a; European Medicines Agency, 2024a; European Medicines Agency, 2017b; European Medicines Agency, 2020b; European Medicines Agency, 2025d; E uropean Medicines Agency, 2025; European Medicines Agency, 2025f)
Tirzepatide	Yes	The EMA states that there is a limited availability of data for patients aged ≥85 years	yes	Based on some trials, a higher frequency of AE, SAE and discontinuation of trial medication due to AE with increasing age is described, especially gastrointestinal symptoms Due to the small number of patients aged ≥85 years, EMA stated that the tolerability in this age group could not be assessed	(European Medicines Agency, 2022c; European Medicines Agency, 2023a; European Medicines Agency, 2025g)
Dapagliflozin	No	-	yes	The EMA cautions that there could be a higher risk for volume depletion in older patients and they are more likely to have an impaired renal function, and treated with antihypertensive or diuretic drugs	(European Medicines Agency, 2012; European Medicines Agency, 2024c; European Medicines Agency, 2019b; European Medicines Agency, 2020c; European Medicines Agency, 2021a; European Medicines Agency, 2022d)
Empagliflozin	No/yes*	The EMA states that based on some trials regarding heart failure, there are beneficial effects for older patients	yes	The EMA cautions that there could be a higher risk for volume depletion in patients aged ≥75 years	(European Medicines Agency, 2014c; European Medicines Agency 2017d; European Medicines Agency, 2022e; European Medicines Agency, 2023b; European Medicines Agency, 2025e)

AE adverse events; SAE, serious adverse events.

* Different statements as there is more than one indication or more than one product with the same drug available.

Especially in older patient the weight reducing activity of the analyzed drugs and the possibility of associated loss of muscle mass (Linge et al., 2024) are important aspects to be considered, for example, with regard to frailty and moreover consequences like falls and fractures. The analyzed trials did not include data about the effects on body composition or loss of muscle mass in older patients. Subgroup examinations using dual energy x-ray absorptiometry were performed in the STEP 1 (semaglutide vs. placebo, n = 140 in total) and SURMOUNT 1 (tirzepatide vs. placebo, n = 160 in total) trials. The data show a greater reduction of total fat mass as well as visceral fat mass compared to the lean body mass in the interventional groups, and an improvement in body composition, however without age-specific analyses (Wilding et al., 2021; Jastreboff et al., 2022; European Medicines Agency, 2025f; European Medicines Agency, 2025g). In some dapagliflozin trials we identified reports on risk fracture, as a surrogate marker for deleterious changes in body composition. These data are inconclusive with the fracture risk being increased with dapagliflozin (European Medicines Agency, 2019b) or similar in both treatment groups (European Medicines Agency, 2020c). For tirzepatide, we identified one source (European Medicines Agency, 2022c) reporting similar fracture incidence in both treatment groups.

The main limitation of this analysis is the incomplete data availability to answer our research questions, which by itself is one of our main findings as well. Overall, we analyzed 106 trial populations. Out of these, for less than half (n = 49) the proportion of patients aged ≥65 years is specified in the sources considered. Less information is available for the older age groups like ≥75 years (n = 37) or ≥85 years (n = 15). Summarizing all available age-stratified data within the included trials is also restricted due to the definition of age groups, as some publications define these subgroups as patients aged >65 years instead of ≥65 years, for example. Based on the observed incomplete reporting of age-stratified data, it is rather difficult to properly describe efficacy and safety of the analyzed drugs in older patients.

When considering older patients, however, we also have to consider that chronological age may not be the primary determinant of health or disease, and that the decision to prescribe GLP1-RA and/or SGLT2-I is based on disease and functional status, and less so on chronological age. However, when analyzing pivotal trials and the regulatory documents (EPAR and SmPC) emerging from the trials results, we have to acknowledge that only chronological age, but not geriatric syndromes, functional status, or geriatric assessment results, are included as inclusion/exclusion criteria and/or outcome variables.

Another limitation is that the analyzed sources were retrieved by only one author. However, we might clearly point to the fact that we have not prepared a systematic review, where this search strategy might not be sufficient. However, the effect is mitigated by the rather small number of sources - the EPAR and the SmPC (Supplementary Tables S1, S2) – on which our trial search is based. Also, the number of trials within these respective sources is small. Based on our search strategy, using the EPAR and the SmPC as the primary source of information, we have identified the trials relevant for European approval and screened these for representation of older patients, and their respective outcomes. Missing information stated in this analysis are not based on the search strategy, but are caused by the lack of information in trial reports. The only possible way to circumvent this limitation and to gather a complete data set on the proportion of older patients in the respective trial populations and age-specific outcomes, would require access to raw data of all trials referenced here.

Due to our search strategy, we cannot exclude the possibility that we missed trials of other kinds specifically addressing age-specific questions which were not part of the approval process. Particularly, the questions related to beneficial or deleterious body composition changes and related health risks such as falls and fractures in older patients may be hidden in trial reports we have not considered due to the fact that they were not relevant for the European approval process.

It is important to emphasize that the central objective of this review was to assess the extent to which older patients were included in the pivotal trials of GLP1-based therapies and SGLT2-I. The inclusion criteria of this review comprise specifically pivotal trials regardless of the included age groups to come to conclusions about the availability or lack of age-stratified results and not about efficacy or safety of these drug classes in older adults in general.

In conclusion, data availability for older patients is insufficient in pivotal trials of liraglutide, dulaglutide, semaglutide, tirzepatide, dapagliflozin, or empagliflozin included in this review. Thus, a proper description of their efficacy and safety in this population is difficult to achieve. Nonetheless, we acknowledge that authors of several recent review articles (Karagiannis et al., 2021; Linge et al., 2024; Chen et al., 2025; Ceasovschih et al., 2025; Bujdei-Tebeică et al., 2025), by means of selective search strategies, did not describe a clinical problematic role of GLP1-RA and SGLT2-I with regard to the older population.
