# Supplementary material for: Availability of age-stratified evidence for GLP1 receptor agonists and SGLT2 inhibitors: an analysis of European public assessment reports with a focus on older patients
Source: Front Pharmacol. 2026 Oct 20;17:1786211. doi: 10.3389/fphar.2026.1786211 (PMC13727555; doi:10.3389/fphar.2026.1786211)
Supplement: Supplementary file 1 [file Table1.docx]

Supplementary Tables

**Table S1.** List of all screened European public assessment reports (EPAR, n=28)

| drug | document identifiers | | version date | access date | references in manuscript |
| --- | --- | --- | --- | --- | --- |
| liraglutide | Victoza: Assessment report | EMEA/379172/2009 | 09-Jul-2009* | 06-Nov-2024 | (European Medicines Agency, 2009) |
|  | Victoza: Assessment report | EMA/857940/2011 | 22-Sep-2011 | 06-Nov-2024 | (European Medicines Agency, 2011) |
|  | Victoza: Assessment report | EMA/CHMP/805158/2013 | 20-Mar-2014 | 06-Nov-2024 | (European Medicines Agency, 2014a) |
|  | Victoza: Assessment report | EMA/CHMP/351673/2016 | 28-Apr-2016 | 06-Nov-2024 | (European Medicines Agency, 2016) |
|  | Victoza: Assessment report | EMA/479764/2017 | 22-Jun-2017 | 06-Nov-2024 | (European Medicines Agency, 2017a) |
|  | Saxenda: Assessment report | EMA/143005/2015 | 11-Feb-2015 | 06-Nov-2024 | (European Medicines Agency, 2015) |
| dulaglutide | Trulicity: Assessment report | EMA/CHMP/524604/2014 | 25-Sep-2014 | 16-Oct-2024 | (European Medicines Agency, 2014b) |
|  | Trulicity: Assessment report | EMA/585102/2019 | 19-Sep-2019 | 28-Jul-2025 | (European Medicines Agency, 2019a) |
|  | Trulicity: Assessment report | EMA/572529/2020 | 17-Sep-2020 | 28-Jul-2025 | (European Medicines Agency, 2020a) |
| semaglutide | Ozempic: Assessment report | EMA/CHMP/715701/2017 | 14-Dec-2017 | 01-Oct-2024 | (European Medicines Agency, 2017b) |
|  | Ozempic: Assessment report | EMA/21773/2022 | 11-Nov-2021 | 16-Oct-2024 | (European Medicines Agency, 2022b) |
|  | Wegovy: Assessment report | EMA/112307/2022 | 11-Nov-2021 | 30-Sep-2024 | (European Medicines Agency, 2022a) |
|  | Wegovy: Assessment report | EMEA/400658/2024 | 25-Jul-2024 | 01-Oct-2024 | (European Medicines Agency, 2024a) |
|  | Rybelsus: Assessment report | EMA/95374/2020 | 30-Jan-2020 | 16-Oct-2024 | (European Medicines Agency, 2020b) |
| tirzepatide | Mounjaro: Assessment report | EMA/791310/2022 | 21-Jul-2022 | 06-Nov-2024 | (European Medicines Agency, 2022c) |
|  | Mounjaro: Assessment report | EMA/539700/2023 | 09-Nov-2023 | 06-Nov-2024 | (European Medicines Agency, 2023a) |
|  | Mounjaro: Assessment report | EMA/14848/2025 | 12-Dec-2024 | 25-Jul-2025 | (European Medicines Agency, 2024b) |
| dapagliflozin | Forxiga: Assessment report | EMA/689976/2012 | 18-Sep-2012 | 11-Nov-2024 | (European Medicines Agency, 2012) |
|  | Forxiga: PRAC assessment report | EMA/PRAC/637349/2016 | 09-Feb-2017 | 11-Nov-2024 | (European Medicines Agency, 2017c) |
|  | Forxiga: Assessment report | EMA/476671/2019 | 28.08.2019* | 11-Nov-2024 | (European Medicines Agency, 2019b) |
|  | Forxiga: Extension of indication variation assessment report | EMA/574301/2020 | 26-Nov-2020* | 11-Nov-2024 | (European Medicines Agency, 2020c) |
|  | Forxiga: Extension of indication variation assessment report | EMA/431093/2021 | 02-Sep-2021* | 11-Nov-2024 | (European Medicines Agency, 2021a) |
|  | Forxiga: Assessment report | EMA/954956/2022 | 15-Dec-2022 | 11-Nov-2024 | (European Medicines Agency, 2022d) |
| empagliflozin | Jardiance: Assessment report | EMA/CHMP/137741/2014 | 20-Mar-2014 | 27-Sep-2024 | (European Medicines Agency, 2014c) |
|  | Jardiance: Assessment report | EMA/11728/2017 | 15-Dec-2016 | 27-Sep-2024 | (European Medicines Agency, 2017d) |
|  | Jardiance: Extension of indication variation assessment report | EMA/CHMP/275072/2021 | 20-Mar-2021 | 27-Sep-2024 | (European Medicines Agency, 2021b) |
|  | Jardiance: CHMP extension of indication variation assessment report | EMA/102340/2022 | 27-Jan-2022 | 30-Sep-2024 | (European Medicines Agency, 2022e) |
|  | Jardiance: CHMP extension of indication variation assessment report | EMA/304328/2023 | 22-Jun-2023 | 30-Sep-2024 | (European Medicines Agency, 2023b) |

*Date first published on the EMA website is stated, if version date is not specified in the document.

**Table S2.** List of all screened Summary of product characteristics (SmPC, n=9)

The SmPC are published as part of the product information of each medicinal product. The product information is a document required for marketing authorization by the European Medicines Agency (EMA) and is available in all official languages of the European Union.

| drug | document identifiers | | version date  (last updated) | access date | references in manuscript |
| --- | --- | --- | --- | --- | --- |
| liraglutide | Victoza | EPAR – Product information | 20-Feb-2025 | 29-Jul-2025 | (European Medicines Agency, 2025b) |
|  | Saxenda | EPAR – Product information | 27-Jun-2025 | 29-Jul-2025 | (European Medicines Agency, 2025a) |
| dulaglutide | Trulicity | EPAR – Product information | 30-Sep-2024 | 29-Jul-2025 | (European Medicines Agency, 2025c) |
| semaglutide | Ozempic | EPAR – Product information | 16-Jul-2025 | 29-Jul-2025 | (European Medicines Agency, 2025) |
|  | Wegovy | EPAR – Product information | 08-Jan-2025 | 29-Jul-2025 | (European Medicines Agency, 2025f) |
|  | Rybelsus | EPAR – Product information | 08-Jul-2025 | 29-Jul-2025 | (European Medicines Agency, 2025d) |
| tirzepatide | Mounjaro | EPAR – Product information | 25-Jun-2025 | 29-Jul-2025 | (European Medicines Agency, 2025g) |
| dapagliflozin | Forxiga | EPAR – Product information | 29-Oct-2024 | 29-Jul-2025 | (European Medicines Agency, 2024c) |
| empagliflozin | Jardiance | EPAR – Product information | 30-Jun-2025 | 29-Jul-2025 | (European Medicines Agency, 2025e) |
